# Risk prediction to inform surveillance of chronic kidney disease in the US Healthcare Safety Net: a cohort study

**DOI:** 10.1186/s12882-016-0272-0

**Published:** 2016-06-08

**Authors:** Yuxiang Xie, Marlena Maziarz, Delphine S. Tuot, Glenn M. Chertow, Jonathan Himmelfarb, Yoshio N. Hall

**Affiliations:** Kidney Research Institute, Department of Medicine, University of Washington, Seattle, WA USA; Department of Biostatistics, University of Washington, Seattle, WA USA; Division of Nephrology, University of California San Francisco and San Francisco General Hospital, San Francisco, CA USA; Division of Nephrology, School of Medicine, Stanford University, Palo Alto, CA USA; Kidney Research Institute, University of Washington, 325 9th Ave, Box 359606, Seattle, WA 98104 USA

## Abstract

**Background:**

The capacity of electronic health record (EHR) data to guide targeted surveillance in chronic kidney disease (CKD) is unclear. We sought to leverage EHR data for predicting risk of progressing from CKD to end-stage renal disease (ESRD) to help inform surveillance of CKD among vulnerable patients from the healthcare safety-net.

**Methods:**

We conducted a retrospective cohort study of adults (*n* = 28,779) with CKD who received care within 2 regional safety-net health systems during 1996–2009 in the Western United States. The primary outcomes were progression to ESRD and death as ascertained by linkage with United States Renal Data System and Social Security Administration Death Master files, respectively, through September 29, 2011. We evaluated the performance of 3 models which included demographic, comorbidity and laboratory data to predict progression of CKD to ESRD in conditions commonly targeted for disease management (hypertension, diabetes, chronic viral diseases and severe CKD) using traditional discriminatory criteria (AUC) and recent criteria intended to guide population health management strategies.

**Results:**

Overall, 1730 persons progressed to end-stage renal disease and 7628 died during median follow-up of 6.6 years. Performance of risk models incorporating common EHR variables was highest in hypertension, intermediate in diabetes and chronic viral diseases, and lowest in severe CKD. Surveillance of persons who were in the highest quintile of ESRD risk yielded 83–94 %, 74–95 %, and 75–82 % of cases who progressed to ESRD among patients with hypertension, diabetes and chronic viral diseases, respectively. Similar surveillance yielded 42–71 % of ESRD cases among those with severe CKD. Discrimination in all conditions was universally high (AUC ≥0.80) when evaluated using traditional criteria.

**Conclusions:**

Recently proposed discriminatory criteria account for varying risk distribution and when applied to common clinical conditions may help to inform surveillance of CKD in diverse populations.

**Electronic supplementary material:**

The online version of this article (doi:10.1186/s12882-016-0272-0) contains supplementary material, which is available to authorized users.

## Background

In the United States (US), progressive chronic kidney disease (CKD) and particularly end-stage renal disease (ESRD) disproportionately affect traditionally underserved groups including racial-ethnic minorities and persons of low socioeconomic means [1–6]. Despite the disproportionate burden of ESRD observed among racial-ethnic minority and low income groups, effective interventions to slow CKD progression and reduce mortality appear to be underutilized in these populations [7, 8].

A central barrier to applying proven therapies in traditionally underserved settings lies in the absence of mechanisms to efficiently monitor and optimize care provided to the nation’s poor and underinsured [9, 10]. Recently, one large safety-net health system has leveraged prediction analytics and data from the electronic health record (EHR) to accurately identify inpatients with specific conditions who are at high risk for subsequent re-hospitalization [11]. Most patients with moderate or severe CKD suffer from multiple chronic conditions and increasingly receive their care from Chronic Disease Management teams [12, 13]. These clinic-based teams typically target patients with specific conditions (e.g., diabetes mellitus, hypertension, chronic viral diseases, congestive heart failure, severe CKD, etc.) and seek to optimize important risk factors for progressive disease, disability and mortality [13, 14]. However, even within these clinic- or disease-based practices most individuals with CKD will not progress to ESRD. Whether data from the EHR can be “meaningfully” used to identify persons at high risk for progressive CKD within this practice-based construct is unclear.

To address this issue, we examined the performance of EHR-based risk predictive models to discriminate among persons with CKD who would and would not progress to ESRD for time frames up to 7 years. We hypothesized that the discriminatory ability (and usefulness) of these models to accurately predict ESRD would vary substantially within clinical subgroups due primarily to differences in patient composition and in the distribution of ESRD risk.

## Methods

### Design, setting, and participants

We conducted a retrospective cohort study of 28,779 persons aged 18 years or older with non-dialysis requiring CKD stages 3–5 who received healthcare in the San Francisco Health Network (SFHN) or Harborview Medical Center (HMC) from January 1, 1996 to December 31, 2009. Eligible subjects included prevalent patients of SFHN or HMC with at least 2 or more ambulatory serum creatinine measurements separated by at least 3 months. We defined CKD stage 3–5 based on the presence of 2 or more serum creatinine measurements yielding an estimated glomerular filtration rate (eGFR) <60 mL/min/1.73 m^2^ as calculated by the re-expressed Modification of Diet in Renal Disease (MDRD) study equation based on calibrated serum creatinine, age, race and sex that were separated by at least 3 months [15, 16]. To reduce the potential for exposure misclassification (i.e., acute kidney injury), we excluded inpatient serum creatinine measurements from consideration. We elected to use the MDRD eGFR (rather than more recently proposed equations) to estimate kidney function because the MDRD eGFR was reported by each health system’s clinical laboratory for most of the study period.

The SFHN (formerly Community Health Network) is the integrated healthcare delivery system of the Department of Public Health of the City and County of San Francisco [7]. During the study period, approximately half of San Francisco’s 130,000 uninsured residents and one-quarter of its Medicaid population made at least one visit annually to the SFHN. Harborview Medical Center in Seattle, Washington, is the Pacific Northwest’s largest provider of care to medically underserved populations, providing over 20 % of all uncompensated care in the state of Washington [17]. Both health systems offer an array of healthcare services including primary care, specialty care and acute care through acute care hospitals (San Francisco General Hospital and Harborview Medical Center) with on-site primary and specialty care clinics, as well as through community-based primary care clinics. Specialty care clinics include diabetes, HIV, hypertension, and nephrology clinics. Services are rendered irrespective of a patient’s ability to pay and further include a wide range of interpreter services to facilitate care for each system’s diverse patient population. Specific details of the health systems have been previously described [18].

### Outcome measures and data sources

The primary outcome measure was progression to ESRD, defined as having a first service date for maintenance dialysis or kidney transplantation. We ascertained ESRD by performing linkage with the United States Renal Data System (USRDS) files based on patient name, date of birth and Social Security number [1]. To ascertain death, we performed identifier matching with the Social Security Administration Master Death files using the same patient identifiers described above [19]. We assessed ESRD and death through September 29, 2011, the last date that data were available for both outcomes at the time of identifier linkage. We defined survival time as time from the first qualifying serum creatinine date until ESRD, death or the end of follow-up through September 29, 2011, whichever occurred first.

### Independent variables

We extracted sociodemographic and clinical variables that we hypothesized might predict progression of CKD to ESRD based on prior studies. These covariates were defined within the 2-year period closest to the index qualifying serum creatinine measurement (used to calculate initial qualifying eGFR). Individual-level sociodemographic covariates included patient age, sex, race-ethnicity (non-Hispanic white, non-Hispanic black, Hispanic, Asian/Pacific Islander, or other), and health insurance coverage (uninsured, Medicaid, Medicare, or commercial insurance) at the time of initial qualifying eGFR. We ascertained comorbid conditions based on established algorithms using discharge diagnostic codes, ambulatory diagnostic codes and procedural codes for diabetes mellitus, hypertension, cardiovascular disease (defined as coronary artery, cerebrovascular, or peripheral vascular disease), chronic viral disease (hepatitis B virus [HBV], hepatitis C virus [HCV], or HIV/AIDS), and drug or alcohol abuse [20–23]. Laboratory covariates included eGFR, hemoglobin, serum albumin, calcium, cholesterol, and phosphorus concentrations, and the presence and severity of proteinuria according to dipstick urinalysis.

### Statistical approach

We summarized the characteristics of the cohort using means (standard deviations) and proportions. We calculated unadjusted incidence rates of ESRD for the full cohort, and for clinical subgroups defined by diabetes mellitus, hypertension, chronic viral diseases (HBV, HCV and/or HIV) and severe CKD (<30 mL/min/1.73 m^2^). We focused on these four subgroups because they represent common conditions frequently targeted by our Chronic Disease Management programs. To approximate our disease-based clinical practice, we did not require the subgroups to be mutually exclusive. Based on prior studies, we tested three proportional hazards regression models to predict progression to ESRD in each subgroup [24–26]. Model 1 included eGFR, dipstick proteinuria, and the “residual” associations with age, sex, and race-ethnicity. To this model, we added health insurance coverage, comorbidities (diabetes mellitus, CVD, hypertension, substance abuse and chronic viral disease) (**model 2**) and additional laboratory variables (serum albumin, calcium, cholesterol and hemoglobin) (**model 3**) [18].

To evaluate the predictive capacity of the models we used 2-fold cross validation, training and validating our models on separate subsets of the data [27]. We divided each cohort into training and validation sets (2/3 and 1/3 of each cohort, respectively). To reduce potential bias caused by excluding patients with missing data, we performed multiple imputation by chained equations with 10 imputations in the training and validation sets separately using the R package ‘mice’ based on observed variables related to the missingness (i.e., missing at random) [28, 29]. We fitted each model to the 10 training sets, and estimated the hazard ratios and the 95 % confidence intervals taking into account the variability associated with the multiple imputation. The baseline hazard function and the estimated coefficients for each model fit to the training set were fixed and applied to the validation set in order to obtain the probability of ESRD-free survival beyond year 1, 3, 5 and 7 for each subject in the validation set. We applied this procedure to each of the 10 imputed training-validation dataset pairs. We then applied the following discrimination and calibration criteria to assess the predictive performance of our model on each subgroup: (1) receiver operating characteristics (ROC) curve and the area under the ROC curve (AUC) [30]; (2) prediction error (PE) [31]; (3) proportion of cases followed (PCF(q)), and (4) proportion of the population needed to be followed (PNF(p)) [32]. PCF and PNF are pragmatic measures of risk concentration that are directly relevant to public health decision-making [32]. The PCF(q) represents the estimated proportion of cases (or events) that would be captured if we followed proportion q of the population at highest risk. PNF(p) represents the estimated proportion of the population at highest risk that would need to be followed in order to capture proportion p of the events. Larger values of PCF and smaller values of PNF indicate better predictive performance [32].

## Results

### Patient characteristics

Consistent with US safety-net healthcare settings, the CKD cohort was young (mean age 60 years) and had a high proportion (59 %) of individuals from racial-ethnic minority groups (Table 1). Among clinical subgroups, the chronic viral disease subgroup was the youngest and had the highest prevalence of substance abuse and mental health conditions as compared with the other three subgroups (Table 1). In contrast, the hypertension subgroup was the oldest and had the highest prevalence of cardiovascular disease. Men comprised a majority of patients with chronic viral disease and severe CKD (eGFR <30 ml/min/1.73 m^2^), and women comprised over half of patients in the hypertension and diabetes mellitus subgroups. Biochemically, the severe CKD subgroup had higher (mean serum) concentrations of phosphorus, and lower concentrations of albumin, calcium and hemoglobin as compared with the other subgroups (Table 1).

**Table 1:** Baseline characteristics of 28,779 patients with moderate or severe chronic kidney disease (stage 3–5) from the healthcare safety net

Characteristics	All	Hypertension	Diabetes mellitus	Chronic viral diseases^a^	Severe CKD^b^
	*N* = 28,779	*n* = 13,525	*n* = 6569	*n* = 5919	*n* = 2108
*Demographics*					
Age, years, mean (sd)	60 (14)	62 (12)	60 (12)	54 (13)	58 (16)
Male, %	48	46	47	61	59
Race or ethnicity, %					
*White*	41	34	32	44	36
*Black*	17	22	22	25	28
*Hispanic*	12	13	16	11	11
*Asian*	22	27	26	17	18
*Other race or ethnicity*	8	3	4	3	6
*Additional Comorbidities, %*					
Cardiovascular disease	28	45	45	32	33
Chronic lung disease	30	37	36	31	31
Substance abuse	23	27	26	45	33
Depression	31	37	38	37	31
*Health Insurance Coverage, %*					
Commercial/employer group	13	10	10	6	8
Medicaid	22	25	26	36	29
Medicare	37	43	41	33	32
Uninsured	29	23	23	25	31
*Laboratory Measures* ^c^					
eGFR^d^, *ml/min/1.73 m* ^*2*^, mean (sd)	49.0 (10.7)	49.0 (10.7)	48.6 (11.0)	48.2 (11.7)	20.1 (7.4)
Dipstick proteinuria					
*None or trace*	62	63	56	57	30
*1+*	19	18	18	22	24
*2+*	12	11	14	13	24
*≥ 3+*	8	8	12	8	23
Cholesterol, mmol/L, mean (sd)	5.1 (1.4)	5.1 (1.3)	5.0 (1.5)	4.7 (1.5)	4.8 (2.1)
Albumin, g/L, mean (sd)	37.8 (6.9)	38.3 (6.9)	37.5 (7.2)	35.9 (8.4)	32.5 (8.9)
Phosphorus, mmol/L, mean (sd)	1.2 (0.4)	1.2 (0.4)	1.2 (0.4)	1.2 (0.4)	1.6 (0.7)
Calcium, mmol/L, mean (sd)	2.3 (0.2)	2.3 (0.2)	2.3 (0.2)	2.2 (0.2)	2.2 (0.3)
Hemoglobin, g/L, mean (sd)	129.3 (20.2)	129.7 (19.1)	127.2 (19.1)	124.2 (23.0)	114.4 (23.4)

Missing values in the full cohort were distributed as follows: dipstick proteinuria, 28.3 %; serum cholesterol, 15.1 %; serum calcium, 5.9 %; serum albumin, 1.9 %; and hemoglobin, 1.8 %. HIV, hepatitis C virus, and/or hepatitis B virus infection

Patients with multiple comorbidities among hypertension, diabetes mellitus, chronic viral diseases and severe CKD are included in each category (i.e., counts for the major comorbid categories listed in the columns above may overlap)

^a^HIV, hepatitis C virus, and/or hepatitis B virus infection

^b^Chronic kidney disease stages 4–5

^c^Laboratory values represent serum concentrations unless noted otherwise

^d^Estimated glomerular filtration rate

### ESRD risk distribution

**Fig. 1:**
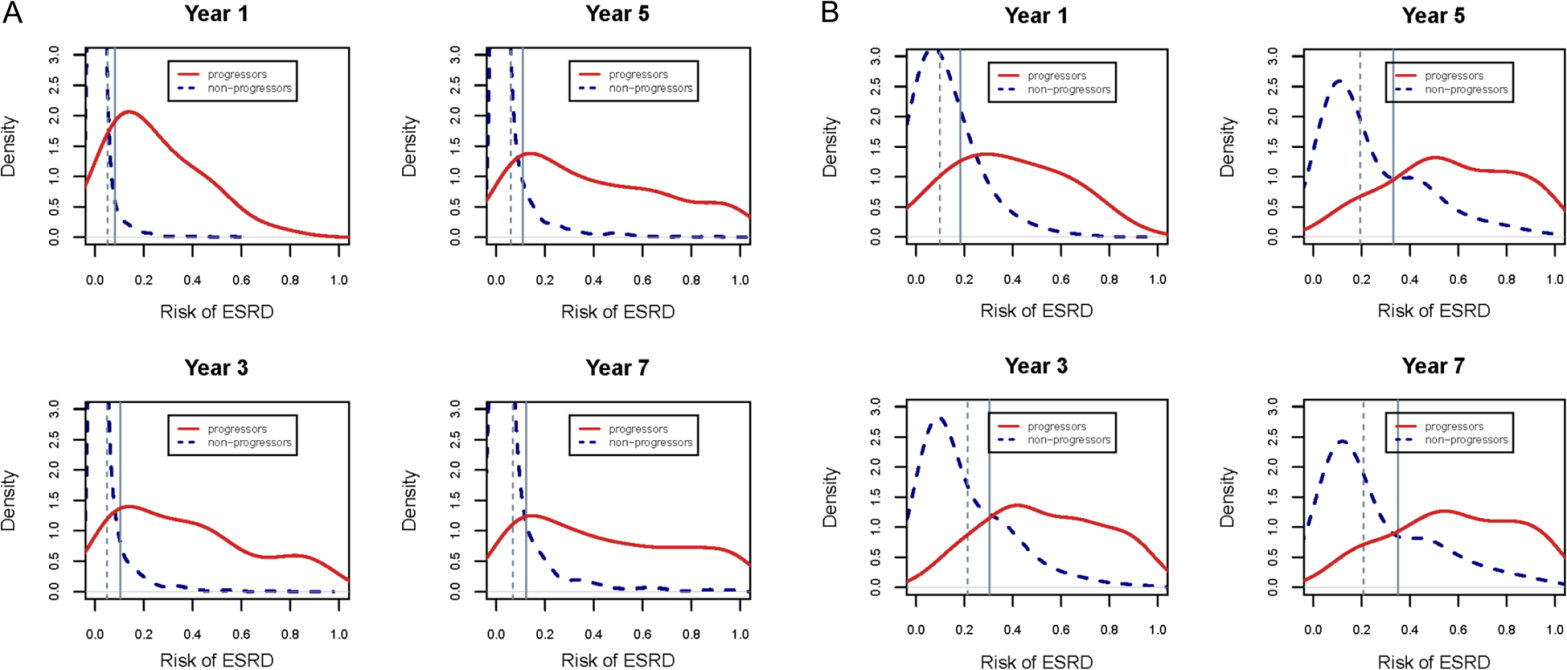
**a** The distributions of predicted risk of ESRD among persons with hypertension. The distributions of predicted risk of ESRD among subjects who did not develop ESRD (non-progressors) in a given time frame is shown by the blue line and subjects who progressed to ESRD (progressors) in that time frame is represented by the red line. We considered four time frames - 1 year, and 3, 5 and 7 years. 80 % of the ESRD progressors are to the right of the vertical solid grey line (q80), and 90 % of them are to the right of the vertical dashed grey line (q90). The risk predictions are based on application of a proportional hazards regression model incorporating age, race, sex, eGFR and dipstick proteinuria to the validation dataset. **b** The distributions of predicted risk of ESRD among persons with severe CKD (eGFR < 30 ml/min/1.73 m^2^). The distributions of predicted risk of ESRD among subjects who did not develop ESRD (non-progressors) in a given time frame is shown by the blue line and subjects who progressed to ESRD (progressors) in that time frame is represented by the red line. We considered four time frames - 1 year, and 3, 5 and 7 years. 80 % of the ESRD progressors are to the right of the vertical solid grey line (q80), and 90 % of them are to the right of the vertical dashed grey line (q90). The risk predictions are based on application of a proportional hazards regression model incorporating age, race, sex, eGFR and dipstick proteinuria to the validation dataset

Figure 1a and b show the estimated risk distribution for ESRD in the hypertension and severe CKD subgroups for time frames up to 7 years using the base model (age, race, sex, eGFR and dipstick proteinuria). In the Figures, 80 and 90 % of the ESRD progressors at highest predicted risk are shown to the right of the vertical solid grey line (q80) and vertical dashed line (q90), respectively. In the hypertension subgroup, the base model discriminated progressors (blue line) from non-progressors (red line) well as reflected by little overlap in their respective risk distribution for q80 and q90. In contrast, substantial overlap in the distribution of ESRD among progressors and non-progressors was observed using the same model in the severe CKD subgroup (Fig. 1b). Similar to hypertension, largely favorable separation in risk distribution was observed in the chronic viral disease and diabetes mellitus subgroups (Additional file 1: Figures S1 and S2).

### ESRD model performance

Overall, 1730 persons initiated ESRD treatment and 7628 died during median follow-up of 6.6 years (Table 2). Persons with severe CKD experienced the highest and persons with hypertension the lowest incidence rates of ESRD among all subgroups (Table 2). Nearly 1 in 3 patients in the severe CKD subgroup progressed to ESRD as compared with approximately 1 in 14 in the hypertension and 1 in 9 in the diabetes mellitus subgroups.

**Table 2:** Incidence rates of end-stage renal disease and death by comorbid subgroup among 28,779 patients with moderate or severe chronic kidney disease from the healthcare safety net

Subgroup	N at risk	Time at risk x1000 person-years		ESRD events	Deaths	ESRD rate (per 1000 person-years)	Death rate (per 1000 person-years)
		ESRD	Death				
All	28,779	198.8	220.5	1730	7628	8.7	34.6
Hypertension	13,525	91.1	101.5	1056	3715	11.6	36.6
Diabetes mellitus	6569	42.8	48.6	804	2060	18.8	42.2
Chronic viral diseases^a^	5919	39.0	41.8	429	2126	11.2	50.9
Severe CKD^b^	2108	11.2	15.6	635	843	56.6	53.9

^a^HIV, hepatitis C virus, and/or hepatitis B virus infection

^b^Chronic kidney disease stages 4–5 (estimated glomerular filtration rate <30 ml/min/1.73 m^2^)

**Fig. 2:**
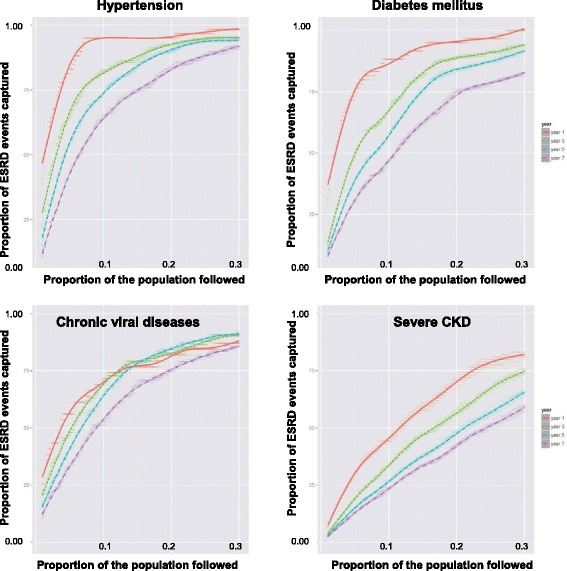
Performance of the base model* for predicting progression of CKD to ESRD in different clinical conditions. The estimated proportion of ESRD events captured (PCF) among a given proportion of subjects at highest estimated risk of ESRD (PNF) for a model* incorporating age, race, sex, eGFR and dipstick proteinuria at 1, 3, 5 and 7-year time frames among persons with hypertension, chronic viral disease, diabetes mellitus and severe CKD

In the full cohort, models that included five common variables (age, race, sex, eGFR, dipstick proteinuria) from the EHR performed well in discriminating progressors and non-progressors, whereby surveillance of individuals in the highest quintile of ESRD risk yielded 97, 91, 86 and 81 % of cases at years 1, 3, 5 and 7, respectively as estimated by PCF(0.2) (Table 3). A predictive model which included these five variables performed similarly well in the hypertension and modestly lower in the chronic viral disease and diabetes mellitus subgroups (Fig. 2). Model performance was substantially lower in the subgroup with severe CKD. Using the base model (age, race, sex, eGFR and dipstick proteinuria), surveillance of individuals in the highest quintile of ESRD risk yielded an estimated 71 % of ESRD events occurring within 1 year in persons with severe CKD. The corresponding PCF(0.2) values declined to 57, 48 and 42 % (ESRD events captured) for the 3-, 5-, and 7-year time frames, respectively (Table 3). Notably, discrimination based on traditional criteria was generally favorable in all subgroups including in severe CKD (AUC, 0.80–0.87) using the same model (note: models with AUC values <0.70, with 0.50 being random and 1.00 being perfect, are considered to have only moderate ability to discriminate risk for an individual patient) [30]. Similar performance patterns and covariate estimates were observed for more complex models 2 and 3 (Additional file 1: Tables S1-S3).

**Table 3:** Comparative performance of the base predictive model* for end-stage renal disease by comorbid subgroup among 28,779 patients with moderate or severe chronic kidney disease from the healthcare safety net

	Measure	All	Hypertension	Diabetes mellitus	Chronic viral diseases	Severe CKD
Year 1	AUC	0.97 (0.01)	0.97 (0.01)	0.97 (0.02)	0.89 (0.03)	0.87 (0.02)
	PE	0.01 (0.00)	0.01 (0.00)	0.02 (0.00)	0.02 (0.00)	0.09 (0.02)
	PCF (0.1)	0.91 (0.03)	0.95 (0.04)	0.88 (0.05)	0.71 (0.06)	0.44 (0.04)
	PCF (0.2)	0.97 (0.02)	0.95 (0.03)	0.95 (0.04)	0.82 (0.05)	0.71 (0.04)
	PNF (0.8)	0.05 (0.02)	0.04 (0.03)	0.07 (0.03)	0.18 (0.07)	0.26 (0.05)
	PNF (0.9)	0.09 (0.05)	0.06 (0.05)	0.13 (0.06)	0.42 (0.12)	0.46 (0.07)
Year 3	AUC	0.94 (0.01)	0.94 (0.01)	0.94 (0.01)	0.91 (0.02)	0.87 (0.02)
	PE	0.02 (0.00)	0.02 (0.00)	0.04 (0.01)	0.04 (0.00)	0.14 (0.04)
	PCF (0.1)	0.83 (0.02)	0.82 (0.03)	0.68 (0.03)	0.71 (0.04)	0.34 (0.02)
	PCF (0.2)	0.91 (0.02)	0.92 (0.03)	0.89 (0.03)	0.83 (0.04)	0.57 (0.03)
	PNF (0.8)	0.08 (0.02)	0.09 (0.02)	0.14 (0.03)	0.17 (0.05)	0.39 (0.04)
	PNF (0.9)	0.17 (0.04)	0.17 (0.06)	0.23 (0.06)	0.26 (0.08)	0.47 (0.05)
Year 5	AUC	0.92 (0.01)	0.94 (0.01)	0.92 (0.01)	0.92 (0.02)	0.83 (0.02)
	PE	0.04 (0.00)	0.04 (0.01)	0.07 (0.01)	0.06 (0.01)	0.20 (0.06)
	PCF (0.1)	0.74 (0.02)	0.73 (0.03)	0.57 (0.03)	0.64 (0.04)	0.27 (0.02)
	PCF (0.2)	0.86 (0.02)	0.90 (0.02)	0.84 (0.02)	0.85 (0.04)	0.48 (0.03)
	PNF (0.8)	0.13 (0.02)	0.13 (0.02)	0.16 (0.03)	0.17 (0.04)	0.45 (0.04)
	PNF (0.9)	0.24 (0.04)	0.20 (0.05)	0.29 (0.05)	0.26 (0.08)	0.57 (0.04)
Year 7	AUC	0.90 (0.01)	0.90 (0.01)	0.88 (0.01)	0.88 (0.02)	0.80 (0.02)
	PE	0.09 (0.01)	0.10 (0.02)	0.15 (0.03)	0.12 (0.02)	0.27 (0.08)
	PCF (0.1)	0.64 (0.02)	0.64 (0.02)	0.47 (0.03)	0.53 (0.04)	0.23 (0.02)
	PCF (0.2)	0.81 (0.02)	0.83 (0.02)	0.75 (0.03)	0.75 (0.04)	0.42 (0.03)
	PNF (0.8)	0.19 (0.02)	0.18 (0.03)	0.27 (0.03)	0.24 (0.05)	0.47 (0.04)
	PNF (0.9)	0.31 (0.04)	0.28 (0.05)	0.38 (0.05)	0.39 (0.07)	0.60 (0.04)

Estimates (standard errors) of the measures of predictive performance of proportional hazards regression model adjusted for age, sex, race-ethnicity, eGFR, dipstick proteinuria and an interaction between eGFR and dipstick proteinuria. The model was fit to each of the ten imputed training sets and evaluated on each of the corresponding validation sets. The ten multiply imputed training sets were based on two-thirds of the dataset (randomly selected, stratified on eGFR), the ten multiply imputed validation sets were based on the remaining one-third of the study data for each subgroup (the imputations were performed separately on the training and validation sets)

*Abbreviations*: *AUC* area under the ROC curve (measure of discrimination); *PE* prediction error (measure of calibration); *PCF*(*q*) proportion of cases followed if proportion q of the population at highest risk is followed; *PNF*(*p*) proportion of the population at highest risk that needs to be followed to capture the proportion p of the cases

## Discussion

Surveillance of “real-world” care delivery to vulnerable groups is challenging because kidney disease metrics are not routinely measured or reported by federally qualified health centers nor are they part of reporting requirements for health plans [33, 34]. Thus, underserved or vulnerable patients with non-dialysis dependent CKD remain invisible to much of the healthcare system unless and until they reach ESRD (at which time most become eligible for Medicare). In this diverse cohort of persons with CKD, we observed that risk predictive models using common data from the EHR can accurately discriminated between most persons who did and did not progress to ESRD for time frames up to 7 years. However, the performance of these risk models varied when applied to specific conditions frequently targeted for Chronic Disease Management. Model performance was highest in the hypertension subgroup, intermediate in chronic viral disease and diabetes mellitus subgroups, and lowest in the subgroup with severe CKD (eGFR < 30 ml/min/1.73 m^2^). Our study findings may help health organizations and their clinical practices to optimize care assessment by estimating the scope and potential needs of patients with CKD who are at highest risk for disease progression, disability and ESRD-related costs.

Health researchers frequently assess risk at the individual level using epidemiological studies or by examining patient-level interventions in randomized clinical trials [24–26]. Tangri et al. evaluated the performance of several risk predictive models based on data extracted from the EHR of 2 CKD cohorts in Canada using traditional discriminatory criteria (C-statistic/AUC and integrated discrimination improvement). They observed that most ESRD risk predictive models performed well in patients with moderate or severe CKD who were referred for nephrology evaluation [24]. Complementary studies in large study cohorts have yielded additional risk predictive models for ESRD which are intended for use by clinicians to estimate individual patient- rather than population-level risk [25, 26]. Using criteria (proportion of cases followed and proportion needed to follow) designed to inform population-level disease assessment, we recently observed that a model incorporating age, race, sex, eGFR and dipstick proteinuria adequately predicted progression to ESRD among vulnerable persons with moderate or severe CKD who were identified through systematic review of the EHR [18]. Our current study findings extend and leverage our prior work, by placing the proportion of cases followed (PCF) and proportion of the population needed to be followed (PNF) more squarely in the context of how clinical care is actually delivered for this patient population. Collectively, our observations suggest that readily available data from the EHR might be efficiently used in earlier stages of CKD to further inform care assessment and planning for organizations or practices based on clinical conditions commonly targeted by disease management programs. For example, our methods could be applied to hypertension clinic to estimate the potential feasibility or effectiveness of an intervention or program targeting of patients in the highest decile or quintile of ESRD risk (e.g., for additional interventions or pragmatic studies, etc.). The resources needed to follow a targeted group of high-risk patients from such disease-based program could be markedly lower than required for risk-stratifying an entire health system. Because CKD represents a heterogeneous array of underlying disease states, this disease- or risk factor-based approach could theoretically leverage existing programmatic resources and infrastructure as an alternative to lumping all high-risk CKD patients into a single category.

In terms of discriminatory assessment, our study illustrates how the PCF and PNF can be more informative than traditional discriminatory criteria such as AUC by providing estimates of risk concentration (for the event of interest) in the study population. If risk is concentrated, such as in the hypertension subgroup, values for traditional discriminatory criteria will typically be large, but the converse is not necessarily true [32]. In other words, as observed in the severe CKD subgroup, values for traditional discriminatory criteria may be favorable even when risk is not concentrated [32, 35]. This observation occurs because the AUC evaluates the model’s ability to discriminate between progressors and non-progressors for all risk thresholds, even those that are not as relevant in clinical practice, such as risks close to zero. In contrast, when using PCF and PNF, we can set the risk threshold at a clinically meaningful level. Thus, desirable values of PCF and PNF are achieved only if risk in the target population is concentrated among a relatively small proportion of individuals (at highest risk). The suboptimal performance of our risk models based on PCF/PNF values among persons with severe CKD likely reflected the broader distribution of ESRD risk in this subgroup. Notably, values for AUC were universally favorable in all subgroups, and hence less informative from the perspective of clinical practice. Reduced variance of influential predictors such as eGFR and dipstick proteinuria among patients with severe CKD (relative to patients from the other subgroups) likely further reduced the predictive capacity of our risk models in this subgroup. While the urinalysis dipstick remains an excellent population-level screening tool for proteinuria, its limited utility in predicting progression to ESRD among patients in later stages of CKD has been previously documented [36]. Thus, models which incorporate additional predictors such urinary albumin-to-creatinine ratio (which was measured only in a small fraction of patients in our cohort) and annual decline in eGFR would likely enhance predictive performance in the setting of severe CKD. In addition, patients with severe CKD are also at markedly higher risk of premature death than those with higher levels of kidney function [20]. The elevated risk of death in this severe CKD subgroup poses additional challenges for predicting ESRD since many (if not all) of the covariates examined are also significantly associated with death.

Historically, low individual-level provider and patient awareness of CKD have reinforced the need to optimize multi-level strategies (at the community, organization, practice, and patient levels) to help assess and manage CKD [18, 37–41]. Our study findings demonstrate the potential usefulness of clinical data from the EHR to provide reliable information for CKD care assessment at the level of the organization and at the level of a disease- or clinic-based practice which might readily generalize to other chronic diseases. Accordingly, clinical practices might leverage the EHR, for example, to identify, triage, and monitor the care of patients at highest risk for progressing to ESRD. As evidenced by the high prevalence of psychiatric conditions, drug and alcohol use in our study cohort, such a multi-level health approach would likely require the consideration of a broader array of health determinants than in conventional health settings. Intervention approaches might thus combine EHR-based risk surveillance with facilitators of care engagement such as assistance with transportation, housing, or health insurance applications, drug or alcohol cessation programs, and mental health co-management. When necessary, our methods might also be applied to refer and track patients at highest risk for imminent (<1 year) ESRD to ensure timely placement of dialysis access, transplant referral, and dialysis education. Recent advancements in software now enable predictive analytics to interdigitate with the EHR, further highlighting the potential “real world” and “real time” application of EHR-derived predictive models [11].

Our study is strengthened by the inclusion of adults with moderate or severe CKD from two large safety-net health systems—populations which traditionally bear a disproportionate burden of ESRD and which may benefit from enhanced CKD surveillance. Our study also includes several limitations. First, while we were able to provide detailed demographic and clinical data, and link our cohort to national registries (to obtain complete or nearly complete capture of treated ESRD and vital status), our study was subject to potential bias from under-ascertainment of comorbidities based on diagnostic and procedural codes. Second, misclassification of CKD and its severity using the MDRD GFR estimating equation may also be operative since the MDRD study equation was derived in a population of mostly white and black patients with moderate-to-advanced CKD, very few of whom had diabetes mellitus. Third, while our study included patients from diverse social and demographic groups, this cohort may not be fully representative of patients who utilize the healthcare safety-net in other geographic locations. In addition, our observations require further validation using external data as our predictive models may perform differently in other populations. Lastly, rather than restricting our study to only patients with complete data (and potentially introducing bias from case deletion), we leveraged multiple imputation under the assumption that missing values carried no information about probabilities of missingness. However, our study results may be potentially biased in the unlikely event that this assumption of ‘missing at random’ (MAR) was violated [29].

## Conclusions

In conclusion, common variables from the EHR can be leveraged to adequately discriminate among most patients with CKD who will and will not progress to ESRD in this safety-net healthcare setting. Recently developed discriminatory criteria may be applied to evaluate the ability of risk predictive models to discriminate between progressors and non-progressors only within a clinically relevant range, and thus, help to inform CKD surveillance at multiple levels in diverse clinical settings.

## Abbreviations

AUC, area under the receiver operating characteristics curve; CKD, chronic kidney disease; ESRD, end-stage renal disease; EHR, electronic health record; eGFR, estimated glomerular filtration rate; HBV, hepatitis B virus; HCV, hepatitis C virus; HIV, human immunodeficiency virus; HMC, Harborview Medical Center; MAR, missing at random; MDRD, Modification of Diet in Renal Disease; PCF, proportion of cases followed; PNF, proportion of the population needed to be followed; ROC, receiver operating characteristics; SFHN, San Francisco Health Network; US, United States; USRDS, United States Renal Data System

## Additional file

Additional file 1: Table S1. Estimates (standard errors) of prediction performance measures for models 2 and 3 in the full cohort and by comorbid subgroup. **Table S2** Estimated hazard ratios and the associated 95 % confidence intervals of the association between each of the covariates and ESRD among subjects with Hypertension (training dataset *n* = 8968) at study entry. These estimates are based on 10 imputed datasets. **Table S3** Estimated hazard ratios and the associated 95 % confidence intervals of the association between each of the covariates and ESRD among those with severe CKD (eGFR < 30 ml/min/1.73 m2) at study entry (training dataset *n* = 1403). These estimates are based on 10 imputed datasets. **Figure S1** The distributions of predicted risk of ESRD among persons with chronic viral disease within 1, 3, 5 and 7 years of cohort entry. **Figure S2** The distributions of predicted risk of ESRD among persons with diabetes mellitus within 1, 3, 5 and 7 years of cohort entry. (DOCX 481 kb)

## References

[1] U.S. Renal Data System, USRDS 2014 Annual Data Report: Atlas of Chronic Kidney Disease and End-Stage Renal Disease in the United States, National Institutes of Health, National Institute of Diabetes and Digestive and Kidney Diseases, Bethesda, MD, 2014. Available at https://www.usrds.org/2014/view/Default.aspx. Last accessed on 8 Jan 2016.

[2] Brancati FL, Whittle JC, Whelton PK, Seidler AJ, Klag MJ (1992). The excess incidence of diabetic end-stage renal disease among blacks. A population-based study of potential explanatory factors. JAMA.

[3] Klag MJ, Whelton PK, Randall BL, Neaton JD, Brancati FL, Stamler J (1997). End-stage renal disease in African-American and white men. 16-year MRFIT findings. JAMA.

[4] Perneger TV, Whelton PK, Klag MJ (1995). Race and end-stage renal disease. Socioeconomic status and access to health care as mediating factors. Arch Intern Med.

[5] Norris K, Nissenson AR (2008). Race, gender, and socioeconomic disparities in CKD in the United States. J Am Soc Nephrol.

[6] Hsu CY, Lin F, Vittinghoff E, Shlipak MG (2003). Racial differences in the progression from chronic renal insufficiency to end-stage renal disease in the United States. J Am Soc Nephrol.

[7] Hall YN, Choi AI, Chertow GM, Bindman AB (2010). Chronic kidney disease in the urban poor. Clin J Am Soc Nephrol.

[8] Hall YN, Rodriguez RA, Boyko EJ, Chertow GM, O’Hare AM (2009). Characteristics of uninsured Americans with chronic kidney disease. J Gen Intern Med.

[9] Radhakrishnan J, Remuzzi G, Saran R, Williams DE, Rios-Burrows N, Powe N, Bruck K, Wanner C, Stel VS, Team C-CS (2014). Taming the chronic kidney disease epidemic: a global view of surveillance efforts. Kidney Int.

[10] Saran R, Hedgeman E, Plantinga L, Burrows NR, Gillespie BW, Young EW, Coresh J, Pavkov M, Williams D, Powe NR (2010). Establishing a national chronic kidney disease surveillance system for the United States. Clin J Am Soc Nephrol.

[11] Amarasingham R, Patzer RE, Huesch M, Nguyen NQ, Xie B (2014). Implementing electronic health care predictive analytics: considerations and challenges. Health Aff (Millwood).

[12] Patel UD, Hernandez AF, Liang L, Peterson ED, LaBresh KA, Yancy CW, Albert NM, Ellrodt G, Fonarow GC (2008). Quality of care and outcomes among patients with heart failure and chronic kidney disease: A Get With the Guidelines -- Heart Failure Program study. Amer Heart J.

[13] Narva AS (2009). Optimal preparation for ESRD. Clin J Am Soc Nephrol.

[14] Tuot DS, Diamantidis CJ, Corbett CF, Boulware LE, Fox CH, Harwood DH, Star RA, Rys-Sikora KE, Narva A (2014). The last mile: translational research to improve CKD outcomes. Clin J Am Soc Nephrol.

[15] Levey AS, Bosch JP, Lewis JB, Greene T, Rogers N, Roth D (1999). A more accurate method to estimate glomerular filtration rate from serum creatinine: a new prediction equation. Modification of Diet in Renal Disease Study Group. Ann Intern Med.

[16] Levey AS, Coresh J, Greene T, Stevens LA, Zhang YL, Hendriksen S, Kusek JW, Van Lente F, Chronic Kidney Disease Epidemiology C (2006). Using standardized serum creatinine values in the modification of diet in renal disease study equation for estimating glomerular filtration rate. Ann Intern Med.

[17] Sheffield JV, Young A, Goldstein EA, Logerfo JP (2006). The public hospital mission at Seattle’s Harborview Medical Center: high-quality care for the underserved and excellence in medical education. Acad Med.

[18] Maziarz M, Black RA, Fong CT, Himmelfarb J, Chertow GM, Hall YN (2015). Evaluating risk of ESRD in the Urban Poor. J Am Soc Nephrol.

[19] Social Security Administration Death Master File, 2014. Available at https://www.ssdmf.com/FolderID/1/SessionID/%7B84B5F850-5A82-428A-AC96-6E167A3FBB40%7D/PageVars/Library/InfoManage/Guide.htm. Accessed on 18 Dec 2015.

[20] Go AS, Chertow GM, Fan D, McCulloch CE, Hsu CY (2004). Chronic kidney disease and the risks of death, cardiovascular events, and hospitalization. N Engl J Med.

[21] Borzecki AM, Wong AT, Hickey EC, Ash AS, Berlowitz DR (2004). Identifying hypertension-related comorbidities from administrative data: what’s the optimal approach?. Amer J Med Qual.

[22] Justice AC, Dombrowski E, Conigliaro J, Fultz SL, Gibson D, Madenwald T, Goulet J, Simberkoff M, Butt AA, Rimland D (2006). Veterans Aging Cohort Study (VACS): Overview and description. Med Care.

[23] Miller DR, Safford MM, Pogach LM (2004). Who has diabetes? Best estimates of diabetes prevalence in the Department of Veterans Affairs based on computerized patient data. Diabetes Care.

[24] Tangri N, Stevens LA, Griffith J, Tighiouart H, Djurdjev O, Naimark D, Levin A, Levey AS (2011). A predictive model for progression of chronic kidney disease to kidney failure. JAMA.

[25] Hallan SI, Ritz E, Lydersen S, Romundstad S, Kvenild K, Orth SR (2009). Combining GFR and albuminuria to classify CKD improves prediction of ESRD. J Am Soc Nephrol.

[26] Gansevoort RT, Matsushita K, van der Velde M, Astor BC, Woodward M, Levey AS, de Jong PE, Coresh J (2011). Lower estimated GFR and higher albuminuria are associated with adverse kidney outcomes. A collaborative meta-analysis of general and high-risk population cohorts. Kidney Int.

[27] van Houweligen H, Putter H (2011). Dynamic prediction in clinical survival analysis.

[28] van Buuren S, Groothuis-Oudshoorn K (2011). mice: Multivariate Imputation by Chained Equations in R. J Stat Softw.

[29] Little RJA, Rubin DB (1987). Statistical analysis with missing data.

[30] Pencina MJ, D’Agostino RB, D’Agostino RB, Vasan RS (2008). Evaluating the added predictive ability of a new marker: from area under the ROC curve to reclassification and beyond. Stat Med.

[31] Gerds TA, Schumacher M (2006). Consistent estimation of the expected Brier score in general survival models with right-censored event times. Biom J.

[32] Pfeiffer RM, Gail MH (2011). Two criteria for evaluating risk prediction models. Biometrics.

[33] National Committee for Quality Assurance: Measuring quality. Improving health care. Available at http://www.ncqa.org/HEDISQualityMeasurement/HEDISMeasures/HEDIS2014.aspx. Accessed 8 Jan 2016.

[34] Tuot DS, Grubbs V (2015). Chronic kidney disease care in the US Safety Net. Adv Chronic Kidney Dis.

[35] Pfeiffer RM (2013). Extensions of criteria for evaluating risk prediction models for public health applications. Biostatistics.

[36] Tonelli M, Muntner P, Lloyd A, Manns BJ, James MT, Klarenbach S, Quinn RR, Wiebe N, Hemmelgarn BR, Alberta Kidney Disease N (2011). Using proteinuria and estimated glomerular filtration rate to classify risk in patients with chronic kidney disease: a cohort study. Ann Intern Med.

[37] Tuot DS, Plantinga LC, Hsu CY, Jordan R, Burrows NR, Hedgeman E, Yee J, Saran R, Powe NR, Centers for Disease Control Chronic Kidney Disease Surveillance T (2011). Chronic kidney disease awareness among individuals with clinical markers of kidney dysfunction. Clin J Am Soc Nephrol.

[38] Tuot DS, Plantinga LC (2011). What patients don’t know may hurt them: knowledge and the perception of knowledge among patients with CKD. Kidney Int.

[39] Tuot DS, Plantinga LC, Hsu CY, Powe NR (2012). Is awareness of chronic kidney disease associated with evidence-based guideline-concordant outcomes?. Am J Nephrol.

[40] Plantinga LC, Boulware LE, Coresh J, Stevens LA, Miller ER, Saran R, Messer KL, Levey AS, Powe NR (2008). Patient awareness of chronic kidney disease: trends and predictors. Arch Intern Med.

[41] Wang V, Maciejewski ML, Hammill BG, Hall RK, Van Scoyoc L, Garg AX, Jain AK, Patel UD (2014). Recognition of CKD after the introduction of automated reporting of estimated GFR in the Veterans Health Administration. Clin J Am Soc Nephrol.

