# Supplementary material for: Risk prediction to inform surveillance of chronic kidney disease in the US Healthcare Safety Net: a cohort study
Source: BMC Nephrol. 2016 Jun 8;17:57. doi: 10.1186/s12882-016-0272-0 (PMC4898308; doi:10.1186/s12882-016-0272-0)
Supplement: Additional file 1: Table S1. — Estimates (standard errors) of prediction performance measures for models 2 and 3 in the full cohort and by comorbid subgroup. Table S2 Estimated hazard ratios and the associated 95 % confidence intervals of the association between each of the covariates and ESRD among subjects with Hypertension (training dataset n = 8968) at study entry. These estimates are based on 10 imputed datasets. Table S3 Estimated hazard ratios and the associated 95 % confidence intervals of the association between each of the covariates and ESRD among those with severe CKD (eGFR < 30 ml/min/1.73 m2) at study entry (training dataset n = 1403). These estimates are based on 10 imputed datasets. Figure S1 The distributions of predicted risk of ESRD among persons with chronic viral disease within 1, 3, 5 and 7 years of cohort entry. Figure S2 The distributions of predicted risk of ESRD among persons with diabetes mellitus within 1, 3, 5 and 7 years of cohort entry. (DOCX 481 kb) [file 12882_2016_272_MOESM1_ESM.docx]

**Table S1.** Estimates (standard errors) of prediction performance measures for models 2 and 3 in the full cohort and by comorbid subgroup.

|  | **Measure** | **All** | | **Hypertension** | | **Diabetes mellitus** | | **Viral disease** | | **Severe CKD** | |
| --- | --- | --- | --- | --- | --- | --- | --- | --- | --- | --- | --- |
|  |  | **Model 2** | **Model 3** | **Model 2** | **Model 3** | **Model 2** | **Model 3** | **Model 2** | **Model 3** | **Model 3** | **Model 4** |
| **Year 1** | **AUC** | 0.96 (0.01) | 0.97 (0.01) | 0.97 (0.01) | 0.98 (0.01) | 0.97 (0.02) | 0.97 (0.01) | 0.89 (0.02) | 0.91 (0.02) | 0.86 (0.02) | 0.88 (0.02) |
|  | **PE** | 0.01 (0.00) | 0.01 (0.00) | 0.01 (0.00) | 0.01 (0.00) | 0.02 (0.00) | 0.02 (0.00) | 0.02 (0.00) | 0.02 (0.00) | 0.09 (0.02) | 0.09 (0.02) |
|  | **PCF(0.1)** | 0.89 (0.03) | 0.90 (0.03) | 0.93 (0.04) | 0.93 (0.04) | 0.88 (0.05) | 0.91 (0.05) | 0.72 (0.06) | 0.72 (0.06) | 0.47 (0.04) | 0.48 (0.04) |
|  | **PCF(0.2)** | 0.95 (0.02) | 0.98 (0.02) | 0.97 (0.03) | 0.97 (0.03) | 0.95 (0.04) | 0.97 (0.04) | 0.87 (0.05) | 0.89 (0.05) | 0.69 (0.04) | 0.68 (0.04) |
|  | **PNF(0.8)** | 0.05 (0.02) | 0.04 (0.02) | 0.05 (0.02) | 0.04 (0.02) | 0.07 (0.03) | 0.06 (0.03) | 0.13 (0.06) | 0.13 (0.06) | 0.32 (0.05) | 0.30 (0.05) |
|  | **PNF(0.9)** | 0.11 (0.04) | 0.10 (0.04) | 0.07 (0.05) | 0.05 (0.04) | 0.12 (0.06) | 0.10 (0.06) | 0.49 (0.12) | 0.33 (0.12) | 0.49 (0.07) | 0.44 (0.07) |
| **Year 3** | **AUC** | 0.94 (0.01) | 0.95 (0.01) | 0.95 (0.01) | 0.95 (0.01) | 0.94 (0.01) | 0.95 (0.01) | 0.92 (0.02) | 0.94 (0.02) | 0.88 (0.02) | 0.88 (0.02) |
|  | **PE** | 0.02 (0.00) | 0.02 (0.00) | 0.02 (0.00) | 0.02 (0.00) | 0.05 (0.01) | 0.04 (0.01) | 0.03 (0.01) | 0.03 (0.01) | 0.14 (0.04) | 0.14 (0.05) |
|  | **PCF(0.1)** | 0.82 (0.02) | 0.86 (0.02) | 0.82 (0.03) | 0.84 (0.03) | 0.69 (0.03) | 0.74 (0.03) | 0.70 (0.04) | 0.71 (0.04) | 0.36 (0.02) | 0.35 (0.02) |
|  | **PCF(0.2)** | 0.92 (0.02) | 0.93 (0.02) | 0.94 (0.02) | 0.93 (0.02) | 0.89 (0.03) | 0.91 (0.03) | 0.91 (0.04) | 0.92 (0.04) | 0.59 (0.03) | 0.57 (0.03) |
|  | **PNF(0.8)** | 0.09 (0.02) | 0.08 (0.01) | 0.08 (0.02) | 0.08 (0.02) | 0.14 (0.03) | 0.12 (0.02) | 0.13 (0.04) | 0.13 (0.04) | 0.33 (0.04) | 0.33 (0.04) |
|  | **PNF(0.9)** | 0.18 (0.04) | 0.14 (0.04) | 0.16 (0.05) | 0.14 (0.05) | 0.23 (0.06) | 0.19 (0.05) | 0.19 (0.08) | 0.19 (0.07) | 0.45 (0.05) | 0.46 (0.05) |
| **Year 5** | **AUC** | 0.93 (0.01) | 0.94 (0.01) | 0.94 (0.01) | 0.94 (0.01) | 0.92 (0.01) | 0.93 (0.01) | 0.93 (0.02) | 0.94 (0.01) | 0.84 (0.02) | 0.85 (0.02) |
|  | **PE** | 0.04 (0.00) | 0.04 (0.00) | 0.04 (0.01) | 0.04 (0.01) | 0.07 (0.01) | 0.07 (0.01) | 0.05 (0.01) | 0.05 (0.01) | 0.19 (0.06) | 0.19 (0.07) |
|  | **PCF(0.1)** | 0.76 (0.02) | 0.77 (0.02) | 0.76 (0.03) | 0.77 (0.03) | 0.58 (0.03) | 0.63 (0.03) | 0.69 (0.04) | 0.71 (0.04) | 0.29 (0.02) | 0.28 (0.02) |
|  | **PCF(0.2)** | 0.87 (0.02) | 0.89 (0.02) | 0.91 (0.02) | 0.92 (0.02) | 0.84 (0.02) | 0.85 (0.02) | 0.92 (0.03) | 0.92 (0.03) | 0.49 (0.03) | 0.49 (0.03) |
|  | **PNF(0.8)** | 0.13 (0.02) | 0.12 (0.02) | 0.12 (0.02) | 0.11 (0.02) | 0.17 (0.03) | 0.16 (0.03) | 0.13 (0.04) | 0.13 (0.03) | 0.42 (0.04) | 0.41 (0.04) |
|  | **PNF(0.9)** | 0.23 (0.04) | 0.21 (0.03) | 0.18 (0.05) | 0.17 (0.05) | 0.29 (0.05) | 0.27 (0.05) | 0.18 (0.07) | 0.18 (0.06) | 0.54 (0.04) | 0.53 (0.04) |
| **Year 7** | **AUC** | 0.92 (0.01) | 0.92 (0.01) | 0.91 (0.01) | 0.92 (0.01) | 0.89 (0.01) | 0.89 (0.01) | 0.91 (0.02) | 0.91 (0.02) | 0.83 (0.02) | 0.83 (0.02) |
|  | **PE** | 0.09 (0.01) | 0.08 (0.01) | 0.10 (0.02) | 0.09 (0.02) | 0.15 (0.03) | 0.14 (0.03) | 0.11 (0.03) | 0.11 (0.03) | 0.25 (0.09) | 0.25 (0.09) |
|  | **PCF(0.1)** | 0.67 (0.02) | 0.68 (0.02) | 0.66 (0.03) | 0.67 (0.03) | 0.48 (0.03) | 0.50 (0.02) | 0.61 (0.04) | 0.61 (0.04) | 0.26 (0.02) | 0.26 (0.02) |
|  | **PCF(0.2)** | 0.83 (0.02) | 0.85 (0.02) | 0.86 (0.02) | 0.86 (0.02) | 0.74 (0.03) | 0.74 (0.02) | 0.85 (0.03) | 0.84 (0.04) | 0.44 (0.03) | 0.44 (0.03) |
|  | **PNF(0.8)** | 0.17 (0.02) | 0.16 (0.02) | 0.15 (0.03) | 0.15 (0.02) | 0.27 (0.03) | 0.26 (0.03) | 0.18 (0.04) | 0.18 (0.04) | 0.45 (0.03) | 0.45 (0.03) |
|  | **PNF(0.9)** | 0.29 (0.04) | 0.28 (0.04) | 0.27 (0.05) | 0.26 (0.05) | 0.38 (0.05) | 0.38 (0.05) | 0.26 (0.07) | 0.24 (0.07) | 0.56 (0.04) | 0.55 (0.04) |

Estimates (standard errors) of the measures of predictive performance of models 2 and 3. Each model was fit to each of the ten imputed training sets and evaluated on each of the corresponding validation sets. The ten multiply imputed training sets were based on two-thirds (n = 19,282 for full cohort) of the dataset (randomly selected, stratified on eGFR), the ten multiply imputed validation sets were based on the remaining one-third (n = 9497 for full cohort) of the Harborview Medical Center (HMC) and SFHN datasets (the imputations were performed separately on the training and validation sets). AUC = area under the ROC curve; PE(x10) = prediction error multiplied by a factor of 10; PCF(q) = proportion of events captured if proportion q of the population at highest risk is followed; PNF(p) = proportion of the population at highest risk that needs to be followed to capture the proportion p of the events.

Model 2: model 1 age, sex, race-ethnicity, eGFR *plus* dipstick proteinuria, an interaction between eGFR and dipstick proteinuria, health insurance coverage, comorbidities (diabetes mellitus, CVD, hypertension, substance abuse and chronic viral disease)

Model 3: model 2 covariates *plus* additional laboratory variables (albumin, calcium, hemoglobin and cholesterol)

**Table S2.** Estimated hazard ratios and the associated 95% confidence intervals of the association between each of the covariates and ESRD among subjects with Hypertension (training dataset n = 8968) at study entry. These estimates are based on 10 imputed datasets.

|  | **Model 1** | **Model 2** | **Model 3** |
| --- | --- | --- | --- |
|  | HR (95% CI) | HR (95% CI) | HR (95% CI) |
| Age (per 10 years) | 0.8 (0.75, 0.85) | 0.72 (0.67, 0.77) | 0.73 (0.68, 0.78) |
| Male vs. female | 1.3 (1.12, 1.52) | 1.27 (1.09, 1.48) | 1.49 (1.27, 1.75) |
| Black vs. white | 2.11 (1.74, 2.55) | 2.17 (1.79, 2.65) | 2.1 (1.73, 2.56) |
| Hispanic vs. white | 1.01 (0.77, 1.32) | 1.1 (0.83, 1.45) | 1.07 (0.81, 1.42) |
| Asian vs. white | 1.41 (1.12, 1.77) | 1.45 (1.15, 1.84) | 1.47 (1.16, 1.86) |
| Other vs. white | 1.73 (1.13, 2.63) | 1.66 (1.08, 2.56) | 1.63 (1.06, 2.5) |
| eGFR mL/min/1.73m^2^ (per 5 units) | 0.67 (0.63, 0.72) | 0.67 (0.62, 0.71) | 0.68 (0.64, 0.73) |
| Albumin dipstick 1+ vs. none or trace | 0.66 (0.3, 1.44) | 0.63 (0.28, 1.38) | 0.58 (0.26, 1.29) |
| Albumin dipstick 2+ vs. none or trace | 1.87 (0.95, 3.71) | 2 (1, 3.99) | 1.77 (0.88, 3.57) |
| Albumin dipstick 3+ vs. none or trace | 4.75 (2.46, 9.15) | 4.46 (2.29, 8.66) | 3.68 (1.88, 7.19) |
| Medicare vs. insured |  | 1.36 (1.05, 1.75) | 1.46 (1.13, 1.9) |
| Uninsured vs. insured |  | 0.73 (0.55, 0.96) | 0.74 (0.56, 0.98) |
| Medicaid vs. insured |  | 1.04 (0.81, 1.34) | 1.03 (0.8, 1.32) |
| Diabetes mellitus |  | 2.07 (1.77, 2.43) | 2.01 (1.72, 2.36) |
| Cardiovascular disease |  | 1.16 (0.99, 1.36) | 1.13 (0.97, 1.33) |
| Substance abuse |  | 1 (0.85, 1.19) | 0.95 (0.8, 1.13) |
| Chronic viral disease |  | 1.13 (0.95, 1.34) | 1.07 (0.9, 1.27) |
| Serum albumin (per 1 g/dL) |  |  | 0.81 (0.72, 0.91) |
| Serum calcium (per 1 mg/dL) |  |  | 1.08 (0.98, 1.19) |
| Hemoglobin (per 1 g/dL) |  |  | 0.89 (0.85, 0.93) |
| Cholesterol (per 10 mg/dL) |  |  | 1.01 (1, 1.02) |
| eGFR x albumin dipstick 1+ | 1.17 (1.07, 1.28) | 1.17 (1.07, 1.28) | 1.17 (1.07, 1.28) |
| eGFR x albumin dipstick 2+ | 1.16 (1.07, 1.25) | 1.14 (1.05, 1.24) | 1.14 (1.05, 1.24) |
| eGFR x albumin dipstick 3+ | 1.16 (1.07, 1.25) | 1.15 (1.06, 1.24) | 1.15 (1.07, 1.24) |

**Table S3.** Estimated hazard ratios and the associated 95% confidence intervals of the association between each of the covariates and ESRD among those with severe CKD (eGFR<30 ml/min/1.73m^2^) at study entry (training dataset n = 1403). These estimates are based on 10 imputed datasets.

|  | **Model 1** | **Model 2** | **Model 3** |
| --- | --- | --- | --- |
|  | HR (95% CI) | HR (95% CI) | HR (95% CI) |
| Age (per 10 years) | 0.95 (0.89, 1.01) | 0.85 (0.79, 0.91) | 0.87 (0.81, 0.94) |
| Male vs. female | 1.09 (0.89, 1.35) | 1.15 (0.94, 1.42) | 1.29 (1.04, 1.6) |
| Black vs. white | 1.7 (1.3, 2.22) | 1.71 (1.3, 2.24) | 1.57 (1.2, 2.05) |
| Hispanic vs. white | 1.82 (1.28, 2.57) | 1.58 (1.1, 2.26) | 1.47 (1.03, 2.1) |
| Asian vs. white | 2.05 (1.54, 2.74) | 1.8 (1.34, 2.43) | 1.66 (1.23, 2.25) |
| Other vs. white | 2.21 (1.35, 3.64) | 1.93 (1.16, 3.21) | 2.02 (1.18, 3.44) |
| eGFR mL/min/1.73m^2^ (per 5 units) | 0.71 (0.58, 0.88) | 0.69 (0.55, 0.85) | 0.71 (0.58, 0.88) |
| Albumin dipstick 1+ vs. none or trace | 0.77 (0.21, 2.81) | 0.61 (0.16, 2.33) | 0.62 (0.16, 2.33) |
| Albumin dipstick 2+ vs. none or trace | 2.28 (0.82, 6.38) | 2.08 (0.71, 6.09) | 1.94 (0.66, 5.68) |
| Albumin dipstick 3+ vs. none or trace | 11.19 (4.34, 28.81) | 9.78 (3.68, 25.99) | 10.23 (3.79, 27.61) |
| Medicare vs. insured |  | 0.99 (0.67, 1.47) | 1 (0.67, 1.5) |
| Uninsured vs. insured |  | 0.6 (0.4, 0.91) | 0.63 (0.42, 0.96) |
| Medicaid vs. insured |  | 0.81 (0.55, 1.2) | 0.89 (0.6, 1.33) |
| Diabetes mellitus |  | 2.07 (1.65, 2.59) | 1.96 (1.55, 2.46) |
| Cardiovascular disease |  | 1.15 (0.9, 1.47) | 1.3 (1.01, 1.68) |
| Hypertension |  | 1.05 (0.83, 1.32) | 1.14 (0.9, 1.44) |
| Substance abuse |  | 0.57 (0.44, 0.74) | 0.64 (0.49, 0.82) |
| Chronic viral disease |  | 0.72 (0.56, 0.92) | 0.66 (0.51, 0.85) |
| Serum albumin (per 1 g/dL) |  |  | 1.31 (1.13, 1.51) |
| Serum calcium (per 1 mg/dL) |  |  | 0.9 (0.8, 1) |
| Hemoglobin (per 1 g/dL) |  |  | 0.85 (0.81, 0.89) |
| Cholesterol (per 10 mg/dL) |  |  | 1.02 (1, 1.03) |
| eGFR x albumin dipstick 1+ | 1.25 (0.92, 1.68) | 1.31 (0.97, 1.79) | 1.3 (0.96, 1.76) |
| eGFR x albumin dipstick 2+ | 1.24 (0.98, 1.58) | 1.27 (0.99, 1.63) | 1.27 (0.99, 1.63) |
| eGFR x albumin dipstick 3+ | 0.98 (0.78, 1.23) | 0.99 (0.78, 1.24) | 0.97 (0.76, 1.22) |

**Figure S1.** The distributions of predicted risk of ESRD among persons with chronic viral disease.

**
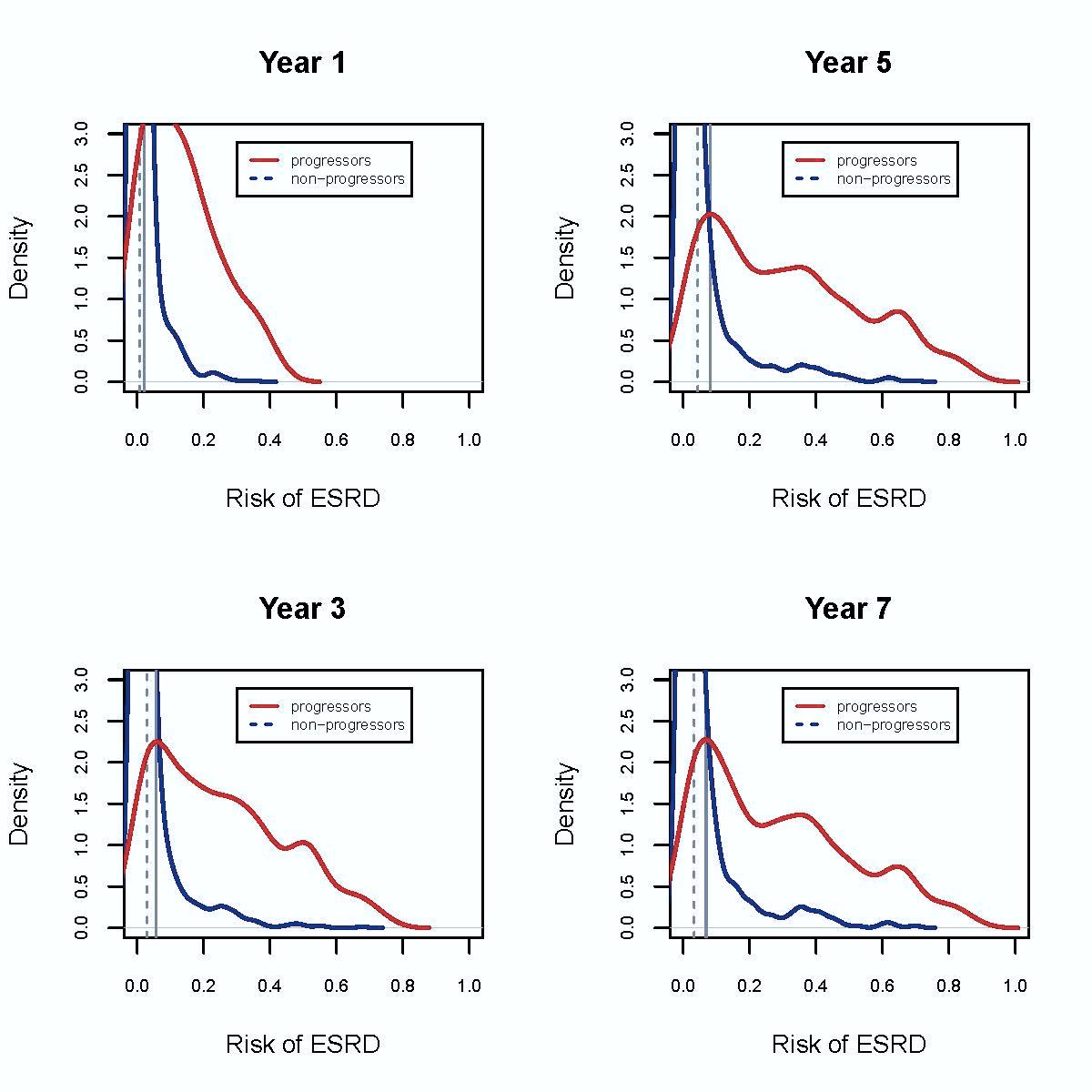
**

**Figure S1 legend.** The distributions of predicted risk of ESRD among subjects who did not develop ESRD (non-progressors) in a given time frame is shown by the dark grey line and subjects who progressed to ESRD (progressors) in that time frame is represented by the light grey line. We considered four time frames - 1 year, and 3, 5 and 7 years. 80% of the ESRD progressors are to the right of the vertical solid grey line (q80), and 90% of them are to the right of the vertical dashed grey line (q90). The risk predictions are based on application of a proportional hazards regression model incorporating age, race, sex, eGFR and dipstick proteinuria to the validation dataset.

**Figure S2.** The distributions of predicted risk of ESRD among persons with diabetes mellitus.

**
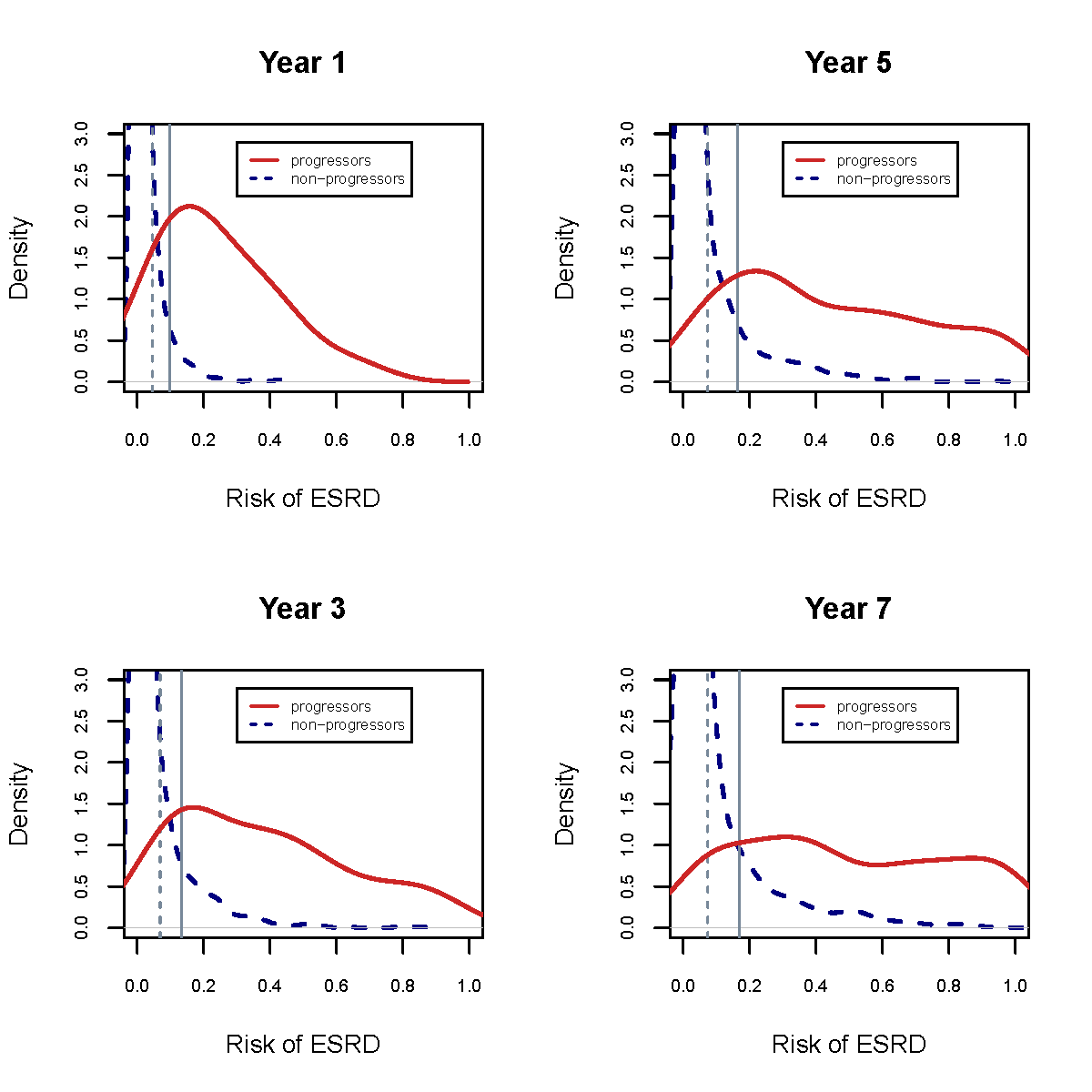
**

**Figure S2 legend.** The distributions of predicted risk of ESRD among subjects who did not develop ESRD (non-progressors) in a given time frame is shown by the dotted line and subjects who progressed to ESRD (progressors) in that time frame is represented by the solid line. We considered four time frames - 1 year, and 3, 5 and 7 years. 80% of the ESRD progressors are to the right of the vertical solid grey line (q80), and 90% of them are to the right of the vertical dashed grey line (q90). The risk predictions are based on application of a proportional hazards regression model incorporating age, race, sex, eGFR and dipstick proteinuria to the validation dataset.
